# Role of Chloride in the Corrosion and Fracture Behavior of Micro-Alloyed Steel in E80 Simulated Fuel Grade Ethanol Environment

**DOI:** 10.3390/ma9060463

**Published:** 2016-06-16

**Authors:** Olufunmilayo O. Joseph, Cleophas A. Loto, Seetharaman Sivaprasad, John A. Ajayi, Soumitra Tarafder

**Affiliations:** 1 Department of Mechanical Engineering, College of Engineering, Covenant University, P.M.B. 1023, Canaanland 112212, Nigeria; akinloto@gmail.com; 2 Materials Science and Technology Division, CSIR-National Metallurgical Laboratory, Jamshedpur 831007, India; shiva@nmlindia.org (S.S.); star@nmlindia.org (S.T.); 3 Department of Chemical, Metallurgical & Materials Engineering, Tshwane University of Technology, Pretoria B-0001, South Africa; 4 Department of Metallurgical and Materials Engineering, School of Engineering and Engineering Technology, Federal University of Technology, P.M.B. 704, Akure 340211, Nigeria; johnadeajayi@yahoo.com

**Keywords:** micro-alloyed steel, fuel grade ethanol, fracture toughness, chloride, pitting corrosion

## Abstract

In this study, micro-alloyed steel (MAS) material normally used in the production of auto parts has been immersed in an E80 simulated fuel grade ethanol (SFGE) environment and its degradation mechanism in the presence of sodium chloride (NaCl) was evaluated. Corrosion behavior was determined through mass loss tests and electrochemical measurements with respect to a reference test in the absence of NaCl. Fracture behavior was determined via J-integral tests with three-point bend specimens at an ambient temperature of 27 °C. The mass loss of MAS increased in E80 with NaCl up to a concentration of 32 mg/L; beyond that threshold, the effect of increasing chloride was insignificant. MAS did not demonstrate distinct passivation behavior, as well as pitting potential with anodic polarization, in the range of the ethanol-chloride ratio. Chloride caused pitting in MAS. The fracture resistance of MAS reduced in E80 with increasing chloride. Crack tip blunting decreased with increasing chloride, thus accounting for the reduction in fracture toughness.

## 1. Introduction

There is currently increasing usage of fuel ethanol as an oxygenate additive in gasoline blends globally [1]. Ethanol has many favorable properties, which makes it preferred for fuel over its fossil counterpart. This includes a higher octane number which influences the anti-knocking property of the fuel, a lower energy yield (close to one third that of petrol) and lower vapor pressure, suggesting less evaporative emissions, amongst others [2]. Fuel ethanol is neither a recent trend, nor one that will die out soon. It is not only a renewable and viable energy source but is non-toxic and so more environmentally friendly than conventional petroleum-based fuels [3,4]. Automobile manufacturers and governments have acknowledged the benefits of using fuel ethanol, and efforts are in progress towards incorporating it into routine use.

However, there have been incidents of corrosion and stress corrosion attacks in some ethanol fuel systems such as zinc-aluminum alloys, carbon steel, castings in fuel pumps and fuel tanks, and welds beside adjacent metal in tank bottoms [5,6]. Several constituents in the fuel that are unsafe for metals under certain conditions result in the corrosive nature of ethanol fuel [5]. Organic acids, peroxides, esters, sulfates, and chlorides are examples of such constituents. Amongst the listed constituents, chlorides act as aggressive ions that may break down passive films on metals, thereby triggering localized corrosion and, in some cases, causing repassivation. Most likely, such film breakdown results from a localized mode of film dissolution due to the adsorption of chloride ions [7]. Reports from several literatures have shown that pitting corrosion (localized corrosion), uniform corrosion and stress corrosion cracking (SCC) are the prevalent mechanisms of corrosion in fuel ethanol systems [5,8,9]. Concerns regarding these corrosion failures and efforts put into preventing them have led to numerous studies on material compatibility with fuel ethanol environments.

A very recent study jointly funded by the American Petroleum Institute (API) and the Renewable Fuels Association (RFA), using the Slow Strain Rate Test method (SSRT), found that SCC of steel can take place in fuel ethanol meeting the ASTM standard D4806 [10]. In addition to water, the most important factor discovered to have caused SCC in fuel ethanol appeared to be dissolved oxygen. When dissolved oxygen minimizes through nitrogen purging, no SCC occurs in the presence of all other species at their maximum levels. Furthermore, reports from another study [11] indicate that the pitting corrosion of carbon steel occurs in simulated fuel grade ethanol (SFGE) solutions. In addition, reports from an evaluation program investigating the SCC behavior of pipeline steel in multiple ethanol environments shows the results of notched-slow strain rate (N-SSR) testing on field samples of fuel grade ethanol (FGE) obtained from Brazilian sources. The crack growth rates determined from N-SSR testing provided an assessment of the severity of cracking. This was also the case with K_ISCC_ (the threshold stress intensity factor for SCC) values based on a fracture mechanics treatment of the N-SSR test data [12]. Fractographic observations reveal that the appearance of cracks caused by other cracking environments is similar to the SCC cracks of steel in fuel ethanol. The cracks are characteristically branched and may possibly be transgranular, intergranular or mixed mode [6].

However, there is currently sparse literature regarding the fracture resistance of steel materials in fuel ethanol environments. It is important to note that most of the SCC tests have been previously conducted using SSR techniques, unlike the application of other techniques like *J*-integral and crack-tip opening displacement (CTOD) tests. The investigation of the SCC mechanism of steel in fuel ethanol is still in the early stages. Since several countries are considering increasing biofuel production as an approach to secure future energy supplies and mitigate global warming, when these come to the market, infrastructure will play a key role in ensuring safe, reliable, and efficient distribution of these fuels to end users [13]. Hence, there is a dire need to evaluate and predict the influence of fuel ethanol on various steel grades that have application in transport and storage systems. Micro-alloyed steel (MAS) has application in the automotive industry and is useful in the fabrication of tanks and fuel storage facilities. This study is, therefore, focused on assessing the influence of chloride on the corrosion and fracture behavior of MAS in E80 simulated fuel ethanol blends.

## 2. Experimental Details

### 2.1. Materials and Test Environments

**Figure 1:**
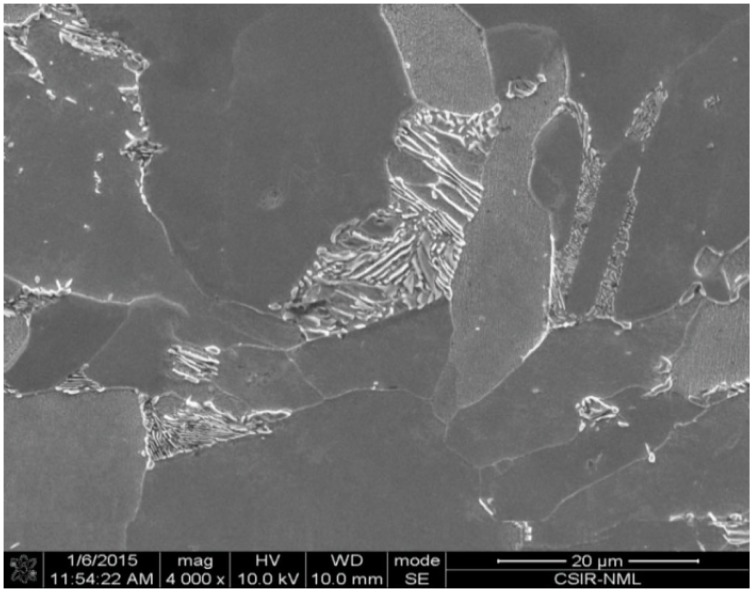
Typical microstructure of MAS in as-received condition showing the presence of ferrite (**dark**) and pearlite (**white**) phases at magnification of 4000×.

The micro-alloyed steel used in this investigation is from commercially produced plates, having the chemical composition shown in Table 1. The microstructure of MAS shows predominantly ferritic structure with pearlite randomly oriented in the ferrite matrix (Figure 1). The fuels used for immersion, electrochemical, and fracture toughness tests include: E80 + 0 mg/L NaCl (as a reference); E80 + 32 mg/L NaCl and; E80 + 64 mg/L NaCl fuel ethanol blends. The test solutions were prepared partly in accordance with the ASTM Standard D4806-01a [14] for fuel grade ethanol. The reagents used include 195 proof ethanol, pure methanol, glacial acetic acid, ultra-pure water (~18 MΩ/cm) and pure sodium chloride (NaCl) with a purity of >99%. NaCl was first dissolved in water, and then added to ethanol to reach the indicated NaCl and water concentrations, respectively. The denaturant used was unleaded gasoline. Table 2 shows the baseline composition for the simulated fuel grade ethanol used in this study. Chloride concentration was systematically altered to study its effect on the corrosion and fracture behavior of micro-alloyed steel. This was achieved by using 0, 32 and 64 mg/L chloride ion (Cl^−^) concentrations. All reagents used were of analytical grade.

### 2.2. Immersion Tests

Flat square coupons with dimensions of 30 mm × 30 mm × 11 mm were machined for the immersion tests. All the specimens were first dry-abraded to 800 grit abrasive paper, then degreased with acetone, dried and used immediately for testing. The area and weight of each specimen was measured before exposure to the test environment for the purpose of post-calculation. The immersion test was carried out for 60 days using duplicate samples for each test condition. Six samples were used for the tests. The samples were suspended with nylon thread in the test solution using airtight plastic containers. In order to minimize changes in solution composition, replace the ionic contaminants, and compensate for evaporation, the test solution was replaced fortnightly. After the immersion tests, thick corrosion products formed on the samples’ surfaces were removed in accordance with the ASTM Standard G1-03 [15] for preparing, cleaning and evaluating corrosion test specimens.

The samples were first cleaned mechanically by scraping off the corrosion products. This was followed by chemical cleaning using Clark’s solution. The solution was prepared with 500 mL hydrochloric acid (specific gravity of 1.19), 10 g of antimony trioxide (Sb_2_O_3_), 25 g of stannous chloride (SnCl_2_) and distilled water. All reagents used were of analytical grade. Cleaning was achieved by stirring vigorously at 25 °C for 5 min. Thereafter, samples were rinsed under running water, cleaned ultrasonically and dried in warm air. The measured final weight subtracted from the initial weight gave the resultant mass loss. An average mass loss was determined from each set of duplicate tests and used to compute the corrosion rates.

### 2.3. Electrochemical Measurements

A Gamry reference 600 Potentiostat/Galvanostat/ZRA (Gamry Instruments, Warminster, PA, USA) was used for open circuit potential (OCP) and anodic polarization measurements. The test setup consists of a three-electrode glass cell with a saturated calomel electrode (SCE) as the reference electrode and a platinum electrode as a counter electrode. The platinum electrode was constructed using Vycor glass. Duplicate experiments were used to evaluate the reproducibility of the test results. Micro-alloyed steel specimens were dry-abraded up to 2000 grit, degreased with acetone, dried and used immediately for testing. The specimens were mounted with bakelite, thus minimizing contact area. The mounted samples were threaded to a carbon steel rod and suspended in solution. A Teflon tape was used to insulate the steel rod from the test solution. The setup was designed in such a way as to keep the distance between electrodes constant for all the tests. The polarization tests commenced with cathodic polarization at −0.25 V *vs.* SCE, with the aim of ensuring a similarly reduced metal surface. In addition, a potential scan rate of 2 mV/s was used to reduce the effect of chloride leakage from the Vycor glass as stated in the literature [16,17].

### 2.4. Visual Examination and Determination of Corrosion Rate

A visual examination was carried out to verify occurrence of pits and discoloration at the end of the immersion tests. The corrosion rate was calculated in milliliters per year using Equation (1) in accordance with ASTM Standard G1-03 [15]:

(1) Corrosion rate=(K×W)/(A×T×D)

where K is a constant (534), *A* is the area in square inches, *T* is the exposure time in hours, W is the mass loss in milligrams, and D is the density in g/cm^3^.

### 2.5. Tensile and Fracture Mechanics Tests

Specimens were fabricated for tensile tests and fracture mechanics tests from the stock materials in as-received condition. The purpose of the tensile tests was to determine the mechanical properties of the as-received micro-alloyed steel material. The fabrication of tensile test specimens was in accordance with ASTM Standard E8M-15a [18]. Round specimens of 5 mm gauge diameter were fabricated from MAS for the tensile tests. The resulting mechanical properties obtained from the room temperature tensile tests are listed in Table 3. The tensile flow curve of MAS exhibited prominent yield point effects.

**Figure 2:**
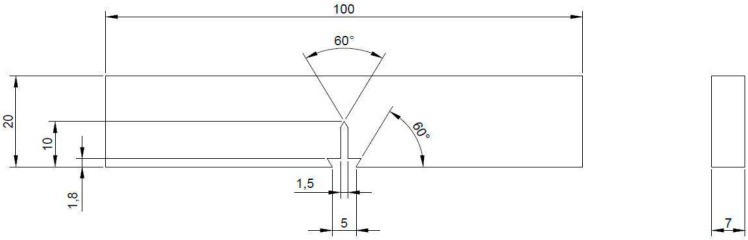
Typical specimen dimension and configuration for three-point bend test (all dimensions in mm).

Monotonic *J*-integral tests were employed for studying the material’s resistance to fracture. To evaluate fracture behavior, three-point bend (TPB) specimens as shown in Figure 2 were employed for carrying out monotonic J-Resistance (*J*-*R*) tests in E80. The nominal width of TPB specimens used was 20 mm. The nominal thickness was 7 mm. Specimens were fabricated by wire-cut electro-discharge machining (EDM) in order to ensure the high levels of precision and alignments demanded for fracture mechanics specimens. Three specimens were used for the tests. All the specimens were equipped with integral knife-edges for compliance-based crack length measurement.

**Figure 3:**
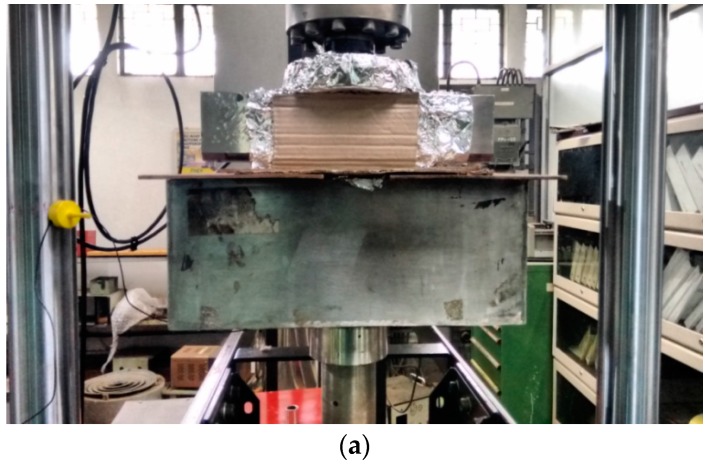

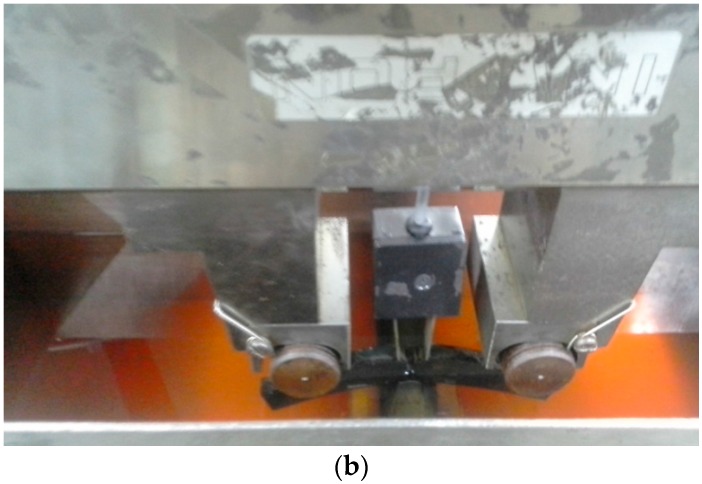
Test Set-up showing (**a**) the covering of the tank to minimize evaporation and (**b**) the sample loaded in three-point bending and the test solution.

Specimens were fatigue pre-cracked under constant ∆*K* of 15 MPa $\sqrt{m}$ and a frequency of 5 Hz with the aid of Instron 8501 servohydraulic testing system interfaced to a computer for test control and data acquisition. Samples were set to pre-crack up to aW=0.5 (where a and W are crack length and width of the specimen, respectively) using a 5 mm COD gauge with a travel of 2 mm. Single specimen fracture toughness tests were carried out according to the procedures laid down in the ASTM Standard E1820 [19]. This standard contains the guidelines for determining elastic-plastic fracture mechanics (EPFM) ductile fracture parameter *J_IC_*. Room temperature tests were performed in a stainless steel test cell as shown in Figure 3 having a total volume of 7 L; 5 L of solution was filled and the vapor space was 2 L. The ramp rate was 10^−4^ mm/s for loading, 10^−2^ mm/s for unloading and 10^−2^ mm/s for reloading. Loading was carried out slowly at the ramp rate of 10^−4^ mm/s in every sequence to heighten SCC effect if any. Consequently, repeat tests were not performed. However, fracture toughness (*J*_0.2_) is derived from the *J*-*R* curve obtained through single-specimen method, according to the procedures laid down in the ASTM Standard E1820. This method is equivalent to performing multiple specimen tests and basically, the single specimen-unloading compliance method has been developed for avoiding the extra burden of testing many specimens and the unnecessary scatter (specimen-to-specimen variation) involved. Nevertheless, the load cell and the COD gauges are calibrated and appropriate alignment maintained to minimize any error in the data.

The calculation of crack lengths was carried out by monitoring the specimen compliance at each instance of unloading. For this purpose, a crack opening displacement (COD) gauge 10 mm in length with 4 mm travel fitted to the specimen load line was used. The tests continued until well below the maximum load bearing capacity of the specimens to guarantee considerable crack extension, and comprised roughly 60 intermediate unloadings. Software was used for test control and data acquisition. The crack length (*a*) at each instance of unloading was computed from the elastic compliance (C) of the unloading curve via the compliance crack length relations as reported elsewhere [20]. The energy parameter J for the instant of *i*th unloading was calculated incrementally using

(2a) $$J_{\left(i\right)}=\frac{{\left(K_{\left(i\right)}\right)}^{2}\left(1-v^{2}\right)}{E}+J_{pl\left(i\right)}$$

where

(2b) $$\;K_{\left(i\right)}=\left[\frac{P_{i}S}{{\left(BB_{N}\right)}^{1/2}W^{3/2}}\right]f\left(a_{i}/W\right)\;$$

and

(2c) $$f\left(a_{i}/W\right)=\frac{3{\left(a_{i}/W\right)}^{1/2}\left[1.99-\left(\frac{a_{i}}{W}\right)\left(1-\frac{a_{i}}{W}\right)\left(2.15-3.93\left(\frac{a_{i}}{W}\right)+2.7{\left(\frac{a_{i}}{W}\right)}^{2}\right)\right]}{2\left(1+2\frac{a_{i}}{W}\right){\left(1-\frac{a_{i}}{W}\right)}^{3/2}}$$

(2d) $$\;J_{pl\left(i\right)}=\left[J_{pl\left(i-1\right)}+\left(\frac{\eta_{pl}}{b_{\left(i-1\right)}}\right)\left(\frac{A_{pl\left(i\right)}-A_{pl\left(i-1\right)}}{B_{N}}\right)\right]\left[1\;-\gamma_{pl}\frac{a_{\left(i\right)}-a_{\left(i-1\right)}}{b_{\left(i-1\right)}}\right]$$

where $\;K_{\left(i\right)}$ is the stress intensity factor calculated from the instantaneous load $P_{i}$ and the crack length $a_{i}$; ν is the Poisson’s ratio; η and γ are geometry and crack-dependent factors; $A_{pl\left(i\right)}$ is the area under the load plastic load line displacement (LLD) curve of the loading displacement; $B_{N}$ is the net specimen thickness, and; $b_{\left(i-1\right)}$ is the incremental remaining crack ligament.

The load, load line displacement and crack tip opening displacement (CTOD) data were processed post-test to obtain *J*-*R* curves. The software incorporated iterative procedures suggested in the ASTM Standard E-1820 [19] for obtaining the experimental blunting line slope *m*, the power law fit of the form $J=C_{1}{\left(\Delta a\right)}^{C_{2}}$ (where $C_{1}$ and $C_{2}$ are constants) constructed to data points within the tearing part of the resistance curve, and the adjusted initial crack length $a_{oq}$. The initiation toughness *J_i_* is the intersection of the blunting line with the power law curve, while *J*_0.2_ signifies the intersection of the 0.2 mm offset blunting line with the power law curve.

### 2.6. Microstructure, Fractography and Physical Characterizations

Characterization of surface morphology after corrosion was carried out using a Field Emission Gun Scanning Electron Microscope (FEG-SEM) coupled with energy dispersive spectrometer (EDS) (FEI, Hillsboro, OR, USA), model FEI-430 NOVA NANO SEM. Fracture surfaces produced through the *J* integral tests were also observed under the FEG-SEM. Corrosion products from immersion tests were analyzed using a Nicolet Almega XR Dispersive Raman spectrometer (Thermo Fisher Scientific, Waltham, MA, USA) having a wavelength of 530 nm.

## 3. Results and Discussion

### 3.1. Effect of Chloride on Mass Loss of MAS in E80

**Figure 4:**
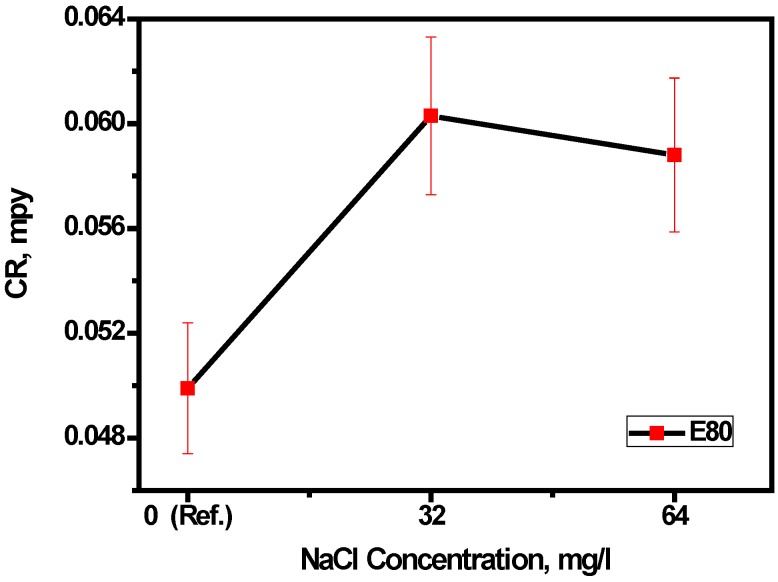
Effect of chloride on the corrosion rate of MAS in E80 simulated fuel ethanol environment. Error bars show standard deviation.

The effects of diverse fuel blends on materials over a period can lead to mass loss. This may be used to project the life cycle of a material in the fuel blends. The procedure for the determination of mass loss and the corresponding corrosion rates from immersion tests have been summarized under the experimental section. In general, the results of duplicate tests were similar, demonstrating the reproducibility of the test technique. The mass loss data for each test were averaged and the corresponding corrosion rates are plotted as shown in Figure 4. The corrosion rate was observed to be lowest (4.99 × 10^−2^ mpy) in the absence of NaCl, whereas the addition of 32 mg/L NaCl increased the corrosion rate drastically to 6.03 × 10^−2^ mpy. A further increase in the concentration of chloride up to 64 mg/L caused a drop in the corrosion rate to 5.88 × 10^−2^ mpy.

The low margin of reduction (approximately 2%) suggests that, beyond the threshold chloride concentration of 32 mg/L, increasing chloride concentration does not have any significant effect on the overall mass loss of MAS. This is confirmed statistically by the ANOVA F-test, as shown in Table 4. ANOVA is a powerful technique for analyzing experimental data involving quantitative measurements. It is useful in factorial experiments where several independent sources of variation may be present [21,22,23]. In this study, a single-factor ANOVA test was used to evaluate the separate and combined effects of varied concentrations of chloride on the corrosion rate of micro-alloyed steel.

As shown in Table 4, the mean-square ratio experimentally determined (1.20) is less than the F ratio (5.46) with 90% confidence. Hence, based on the above test data, it can be concluded with 90% confidence that there is no significant difference between the effects of the three chloride concentrations on the corrosion rate of MAS. This study is significant to the overall field, from the perspective that the determined corrosion rates of MAS in E80 are typically low and generally below the level of normal engineering significance for usage and storage applications as reported in the literature [6]. Therefore, micro-alloyed steel can be supposed to be compatible with an E80 fuel ethanol environment from the standpoint of corrosion.

**Figure 5:**
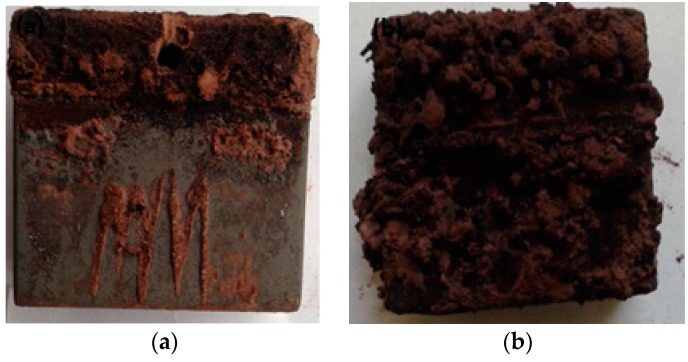
Visual appearance of MAS exposed to ethanol fuels at 27 °C after 60 days. (**a**) E80 + 0 mg/L NaCl; and (**b**) E80 + 32 mg/L NaCl showing increased rust in the presence of chloride.

Figure 5 shows the visual appearance of MAS samples after 60 days of exposure to E80 with and without chloride. In Figure 5b, more rust is seen on MAS after immersion tests in E80 with 32 mg/L chloride, in comparison with the appearance of MAS in Figure 5a. The increase in rust is caused by a substantial increase in oxygen solubility due to the presence of chloride [24].

**Figure 6:**
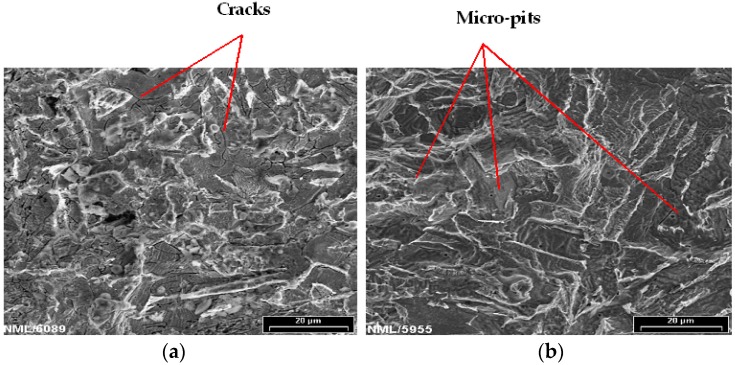
Post-corrosion scanning electron microscope (SEM) images of MAS at 1000× after 60 days immersion in (**a**) E80 + 0 mg/L NaCl with cracks present; and (**b**) E80 + 32 mg/L NaCl showing micro-pits.

Figure 6a shows the scanning electron microscope (SEM) image of the morphology of MAS after cleaning of corrosion products from samples immersed in E80 without chloride for 60 days. Micro-cracks are visible, as indicated with red arrows. In the presence of 32 mg/L NaCl (Figure 6b), micro-pits were formed on the surface of the material. The pitting attack is small at the surface and was not discovered by visual examination since corrosion products covered the samples [25]. The presence of micro-pits is ascribed to the action of Cl^−^ ions in the breakdown of passive surface films formed on MAS in E80, as no pitting was noticed on the samples tested in the absence of chloride. The growth of these pits may lead to perforation and eventually bring about stress corrosion cracking, thereby decreasing the lifespan of MAS. Furthermore, pitting corrosion on MAS in the presence of chloride as observed in this study is in agreement with results of published literatures on the role of chloride in the ethanol corrosion of steel [1,5,17].

### 3.2. Effect of Chloride on Polarization Behavior of MAS in E80

**Figure 7:**
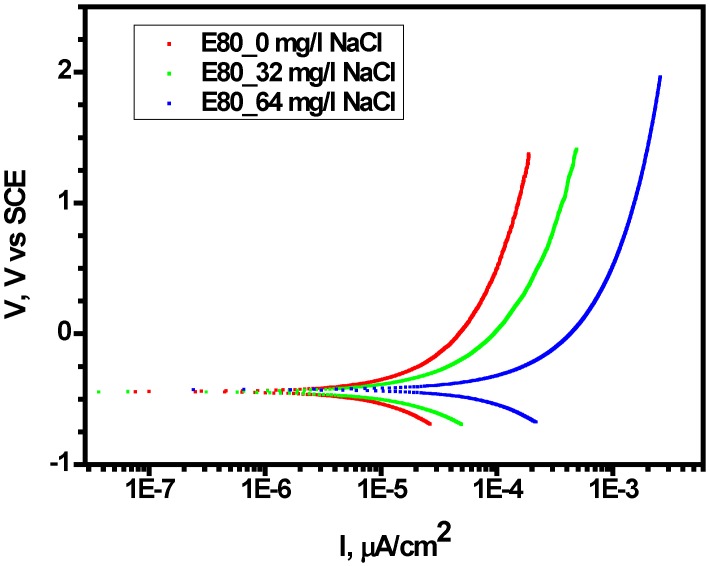
Effect of chloride on anodic polarization of MAS showing increase in current density with increasing chloride in E80.

The polarization behavior of MAS was investigated using anodic polarization via cyclic potentiodynamic polarization, as described in Section 2.3. Figure 7 shows reference electrochemical tests carried out in the absence of chloride and used as a basis for examining the influence of chloride on polarization behavior of MAS. The MAS samples were anodically polarized with similar potential difference (1.5 *V*_SCE_) from their primary OCPs as described elsewhere [17]. A scan rate of 2 mV/s was used for the experiments. Furthermore, Table 5 shows the corrosion potential (*E*_corr_) and estimated current density (*i*_corr-estimate_) as recorded for all the test conditions. Higher current densities were obtained in the existence of chloride at 32 mg/L with respect to the reference test.

Consistent with the results of mass loss tests, the *i*_corr-estimate_ and corrosion rates calculated from tafel slopes increased in 32 mg/L NaCl and decreased in 64 mg/L NaCl. It is evident that MAS did not demonstrate distinct passivation behavior as well as pitting potential with anodic polarization in the range of the ethanol-chloride ratio.

### 3.3. Characterization of the Oxide Layers Formed on MAS Exposed to E80

**Figure 8:**
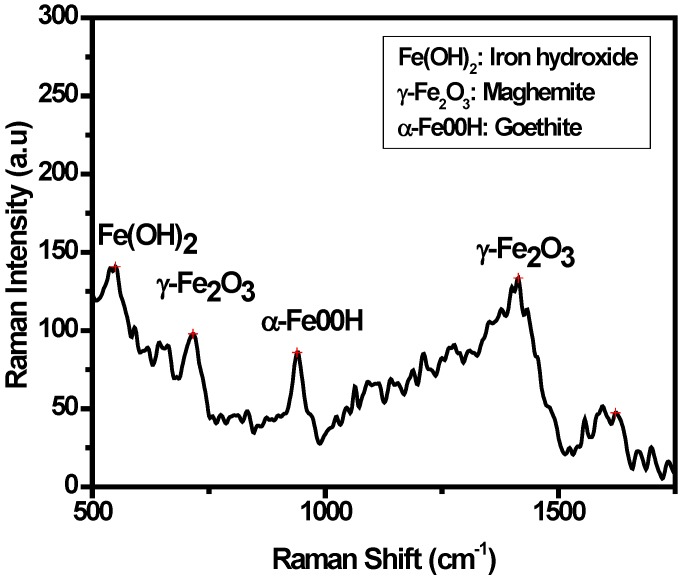
Raman shifts of corrosion products from MAS in simulated E80 fuel ethanol showing the presence of iron hydroxide, maghemite and goethite.

The oxide layers formed on the corroded steel were analyzed by Raman spectroscopy after 60 days of immersion in E80 at 27 °C. The Raman spectrum generated was used to identify and verify the unknown chemical species present in the corrosion products. Changes in frequency shift due to the chemical species were also noted. Figure 8 shows the Raman spectra obtained by analyzing the corrosion products in both the presence and absence of chloride. The presence of iron oxyhydroxides such as goethite [α-FeOOH], iron hydroxide [Fe(OH)_2_], and maghemite [γ-Fe_2_O_3_] are observed in test environments with NaCl [26,27,28]. A strong band at 549 cm^−1^ is seen representing the existence of hematite. The presence of water in the simulated fuel ethanol environments stimulates the formation of iron hydroxide, as described elsewhere [16]. A wide and stronger band at 1423 cm^−1^ present in the Raman spectroscopy of corrosion products obtained from samples immersed in the presence and absence of NaCl shows the presence of maghemite. A strong band of Goethite is likewise present at 550 cm^−1^.

### 3.4. Effect of Chloride on Fracture Behavior of MAS in E80

In studying the fracture behavior of MAS in E80, Elastic-Plastic Fracture Mechanics (EPFM) tests were carried out using the monotonic *J*-integral test method. The overall objective of the test was to develop a load (P)-displacement (V) record that can be used to evaluate fracture toughness (*J*_0.2_) of MAS in an E80 test environment. The test and data analysis procedures are described in detail in Section 2.5. In this section, the influence of chloride ion concentration on the load-displacement plots, fracture toughness (*J*_0.2_) and widths of stretch zones are shown and discussed.

#### 3.4.1. Effect of Chloride on the Load-Displacement Plots of MAS

**Figure 9:**
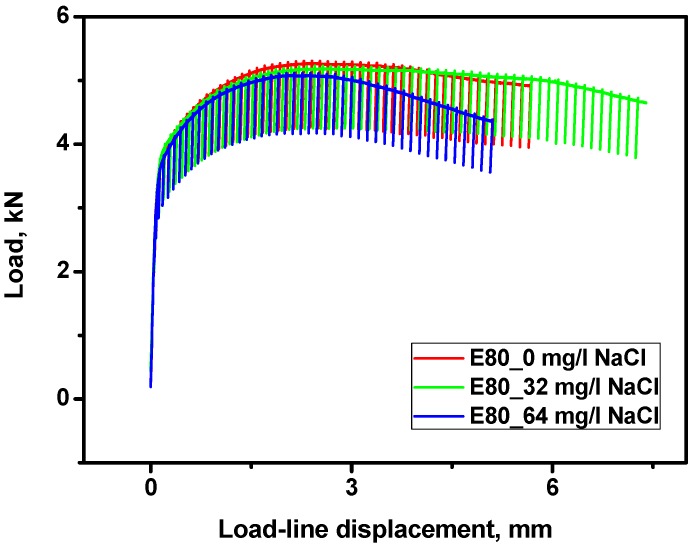
Typical Load-Displacement plots of MAS in E80 with 0, 32, and 64 mg/L NaCl.

Typical load-displacement records obtained from the J-Resistance *J*-*R* test are shown in Figure 9. MAS material showed substantial deformation and significant ductile tearing prior to reaching maximum load, irrespective of the various concentrations of NaCl (0, 32 and 64 mg/L) used in the test environment. The presence of chloride caused no significant deviation from ductile tearing. This is because of the high toughness linked with low strength, as well as the high ductility of MAS.

#### 3.4.2. Effect of Chloride on Fracture Toughness of MAS

**Figure 10:**
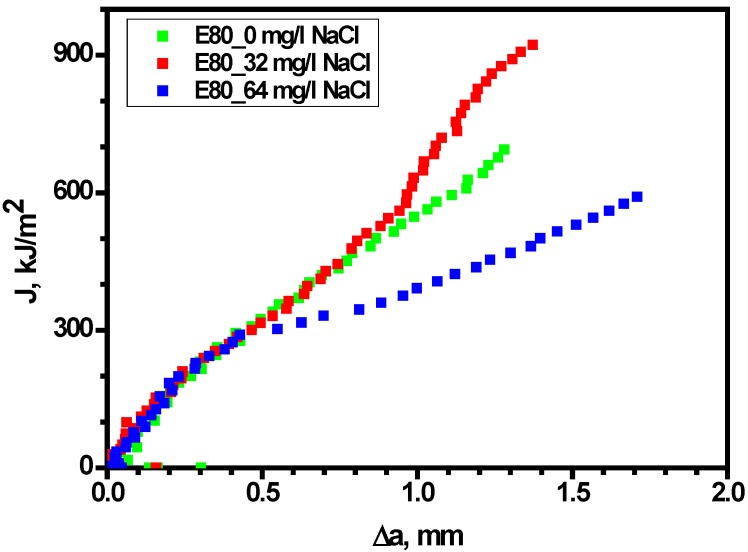
*J*-*R* curves obtained from MAS specimens in E80.

Fracture tests carried out in the presence and absence of chloride revealed close a similarity in the *J*-*R* curves of MAS in E80 with 0 and 32 mg/L NaCl, respectively (as shown in Figure 10). On the other hand, the *J*-*R* behavior of MAS was slightly altered by a decrease in the *J*-*R* curve after the crack initiation in the presence of 64 mg/L NaCl. However, it is more suitable to base evaluations on the critical fracture toughness of the specimens, as reported in the literature [29].

**Figure 11:**
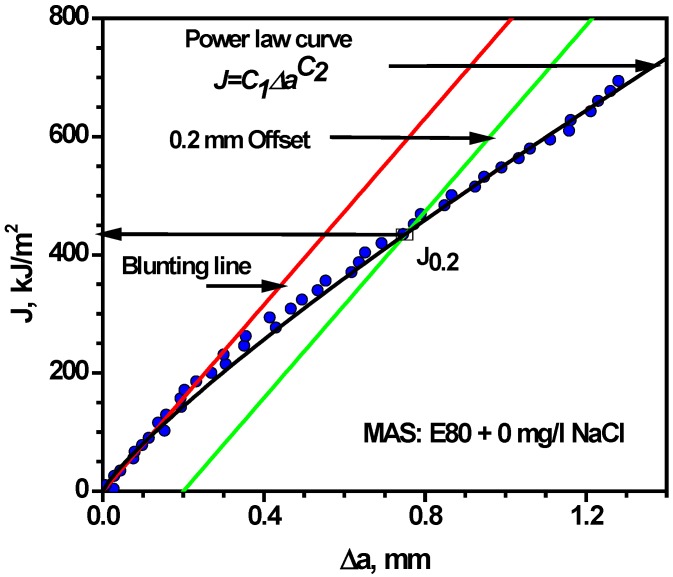
Identification of critical fracture toughness (*J*_0.2_) on the *J*-*R* curve obtained from MAS specimen.

**Figure 12:**
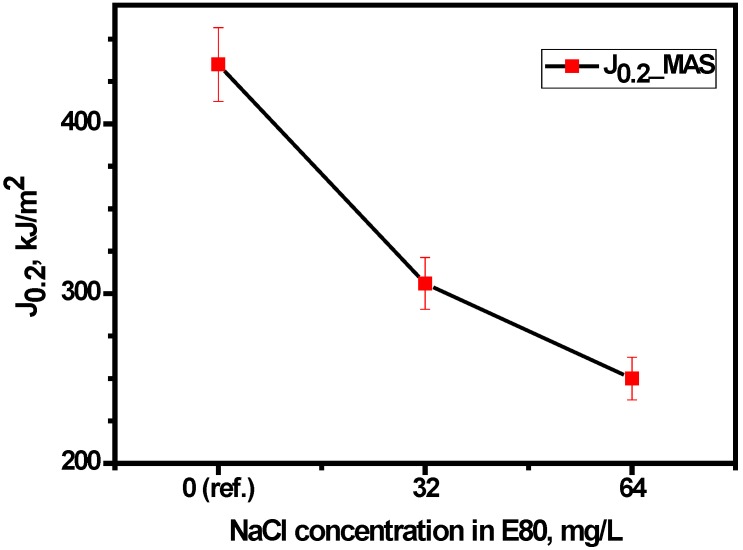
Variation of critical fracture toughness, *J*_0.2_ with test environment.

Consequently, the ASTM E-1820 procedure was used to determine the initiation toughness, *J_i_* and the (unqualified) critical fracture toughness at the 0.2 mm ductile crack extension (*J*_0.2_) through the definition of a best–fit blunting line and the use of a power law curve to outline the tearing region. *J*_0.2_ was estimated to be 435 kJ·m^−2^ for MAS in E80 without chloride. Upon the introduction of 32 mg/L NaCl, *J*_0.2_ decreased to 306 kJ·m^−2^ and 250 kJ·m^−2^ at E80 + 64 mg/L NaCl. The percentage reduction in fracture toughness spanned from 30% to 43% in 32 mg/L NaCl and 64 mg/L NaCl, respectively. This decrease is considered significant with increasing chloride concentration. Figure 11 shows the identification of *J*_0.2_ on a *J*-*R* curve for a specific case of MAS specimen, as per the procedure of the ASTM Standard E-1820. In addition, Figure 12 shows the variation of fracture toughness *J*_0.2_ with the test environment for MAS. Tests carried out with the three concentrations of chloride indicated that the maximum degenerating effect on the fracture toughness of MAS was obtained with 64 mg/L NaCl. The fracture resistance of MAS decreased with respect to the reference test (in the E80 + 0 mg/L NaCl test environment). It is therefore apparent that chloride in the ethanolic solution resulted in decreasing fracture resistance of the material.

To qualify *J*_0.2_ as the ductile fracture toughness *J_Ic_*, the criteria in Equations (3)–(5) have to be satisfied.

(3) $$B>10J_{Q}/\sigma_{o}$$

(4) $$b_{o}>10J_{Q}/\sigma_{o}$$

(5) $${\frac{dJ}{da}|}_{\Delta a_{Q}}<\sigma_{o}$$

where $\sigma_{o}$ is the flow stress and $\Delta a_{Q}$ is the crack extension at *J*_0.2_.

Table 6 shows that, amongst all the values of *J*_0.2_ obtained for MAS specimens in E80, only the fracture toughness of the sample tested in E80 + 64 mg/L NaCl is qualified to be termed the plane strain fracture toughness (*J_IC_*). This means that for the other specimens, the determined fracture toughness values are size-dependent and so amenable to comparisons only with specimens having similar size.

The physical cause behind the decrease in fracture resistance of the micro-alloyed steel exposed to monotonic loading in E80 is further explained with stretch zone measurements.

#### 3.4.3. Effect of Chloride on Widths of Stretch Zones

From a fracture mechanics point of view, the decrease in fracture toughness can be said to originate from the blunting behavior of cracks in MAS upon loading. Crack extension by void coalescence is preceded by the expanse of stretch zone, which is a featureless region immediately after the fatigue pre-crack region. The stretch zone is essentially described as an imprint of the initiation fracture regime of ductile materials, and thus has a correlation with the initiation fracture toughness of a material [30,31]. Numerous attempts have been made to measure stretch zone dimensions and acquire a suitable correlation with ductile fracture toughness [30,31,32,33]. Two stretch zone components are typically associated with extremely ductile materials, namely stretch zone width (*SZW*) and stretch zone depth (*SZD*). However, there is no agreement as regards which of the stretch zone measurements ought to be used for defining critical fracture toughness. Consequently, SZW is used for this study.

**Figure 13:**
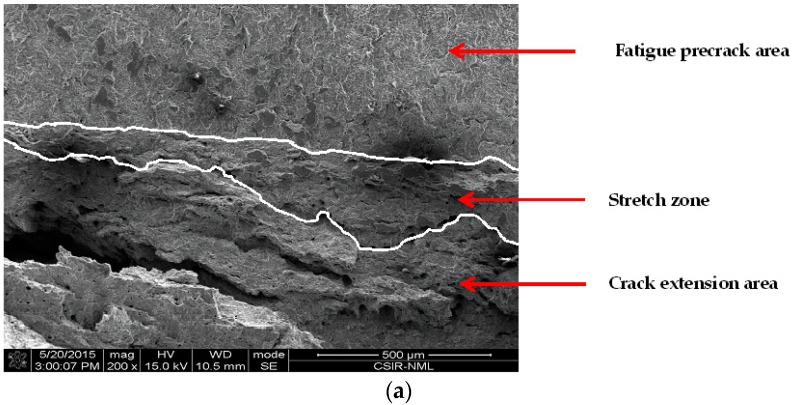

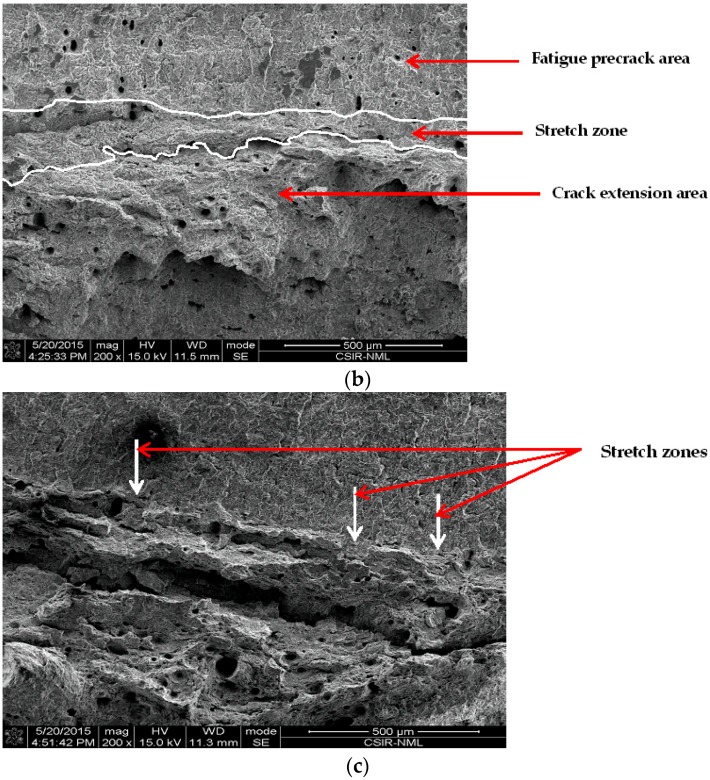
SEM fractograph of *J*-integral tested MAS in E80 with (**a**) 0 mg/L; (**b**) 32 mg/L; and (**c**) 64 mg/L NaCl, showing decreasing stretch zone (SZ) and void coalescence ahead of fatigue pre-crack with increasing chloride.

The values of *J_i_* obtained from the fracture mechanics tests were compared with *SZW* measurements. Figure 13 shows the SEM images of stretch zones on tested MAS fracture surfaces. It is important to note that for all the chloride concentrations, the stretch zones were clearly identified. The fractographs were obtained at a magnification of 200×. The boundaries of the stretch zones were delineated manually to enable measurement. As shown in Figure 13, the crack tip blunting (stretch zone) decreases as chloride concentration increased. The decreased blunting of the crack tip explains the decreasing *J*_0.2_ obtained from the experimental data with increasing chloride. Since there is reduced plastic deformation at the crack tip due to chloride, a smaller amount of energy is therefore required to create a new crack surface. As a result, fracture toughness decreases. At higher chloride concentration of 64 mg/L, blunting was identified in very few locations at the crack front as depicted by the arrows; hence, measurement was not possible for this case. For the fractographs in Figure 13a,b, the *SZW* along the crack front is not even; as a result, several measurements were obtained for each fractograph and the average value computed.

**Figure 14:**
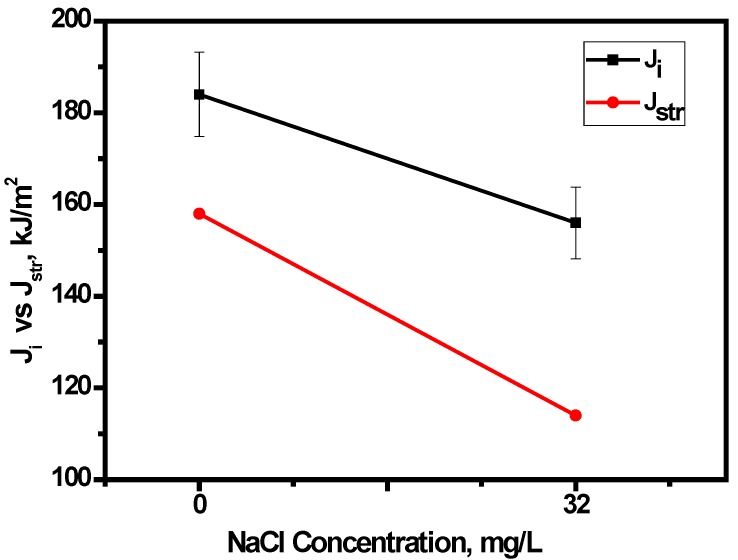
Variation of *J_i_* and *J_str_* of MAS specimens with E80 test environment.

In addition, the micron marker on the SEM image was measured in mm, and the number of microns corresponding to 1 mm was calculated. The measured *SZW* in mm was converted to microns and the fracture initiation toughness was evaluated from the *J*-*R* curves by the vertical intercept at Δa=SZW. The initiation fracture toughness determined from the SZW is denoted as *J_str_* and is plotted as a function of chloride concentration in Figure 14.

It is evident that *J_str_* showed a decreasing trend with increasing chloride concentration. The nature of variation of *J_str_* with chloride concentration in E80 is also similar to that of *J_i_*. Thus, this variation of the nature of *J_str_* with the test environment strongly qualifies the use of *SZW* for determining the fracture toughness of MAS in E80.

## 4. Conclusions

This paper has presented an investigation on corrosion and fracture behavior of micro-alloyed steel in simulated E80 fuel ethanol under the action of 0, 32 and 64 mg/L NaCl concentrations. The explicit conclusions reached are as follows:

(1) The mass loss of MAS increased in the presence of chloride up to a threshold concentration of 32 mg/L. The ANOVA test further confirms, at 90% confidence, that there is no significant difference between 0, 32 and 64 mg/L NaCl concentrations on the corrosion rate of MAS.
(2) Chloride caused pitting in MAS after immersion in E80 with chloride. In the absence of chloride, there was no pitting.
(3) MAS did not demonstrate distinct passivation behavior as well as pitting potential with anodic polarization in the range of the ethanol-chloride ratio.
(4) The fracture resistance of MAS reduced in E80 with increasing chloride and with respect to tests in the absence of chloride.
(5) Crack tip blunting decreased with increasing chloride, thus accounting for a reduction in fracture toughness. In addition, the nature of the variation of *J_str_* with the chloride concentration in E80 is similar to that of *J_i_*, which therefore qualifies the use of *SZW* in determining the initiation fracture toughness of MAS in E80.

## Figures and Tables

**Table 1:** Chemical composition of as-received micro-alloyed steel (MAS) (wt %).

Element	C	Mn	Si	Cr	Ni	Al	Ti	Mo	Cu	Fe
Micro-alloyed	0.13	0.77	0.012	0.027	0.015	0.042	0.0025	0.0017	0.006	balance

**Table 2:** Baseline composition of simulated fuel ethanol based on ASTM D4806 [14].

Ethanol (Vol %)	Methanol (Vol %)	Water (Vol %)	NaCl (mg/L)	Acetic Acid (mg/L)
98.5	0.5	1	32	56

**Table 3:** Mechanical properties of MAS in as-received condition.

Sample	σ_YS_ (MPa)	σ_UTS_ (MPa)	*e_u_* (%)	*e_T_* (%)	n^#^	Log k	H_v_*
MAS	301.54	458.83	18.27	38.89	0.13	2.52	111.8

H_v_* denotes average Vickers hardness obtained from seven readings; n^#^ from σ *= k*ɛ*^n^* where *n* is the strain-hardening exponent; Log k: strength coefficient; *e_u_*: uniform elongation; *e_T_*: total elongation; $\sigma_{o}$: flow stress; σ_YS_: yield stress; σ_UTS_: ultimate tensile stress.

**Table 4:** Analysis-of-variance table for effect of chloride concentration in corrosion rate of MAS.

Source of Variation	Sum of Squares	Degree of Freedom	Mean Square	Mean Square Ratio (MSR)	Min. MSR at 90% Confidence
Chloride concentration	83,041.44	2	41,520.72	1.20	5.46
Residual	103,408.71	3	34,469.57		
Total	186,450.15	5			

**Table 5:** Anodic polarization data showing the influence of chloride in simulated E80 fuel ethanol environment.

Solution Chemistry	*E*_corr_ (mV)	*i*_corr-estimate_ (A/cm^2^)	Corrosion Rate (mpy)
E80 + 0 mg/L NaCl	−3.93 × 10^2^	7.99 × 10^5^	3.56 × 10^1^
E80 + 32 mg/L NaCl	−4.13 × 10^2^	8.27 × 10^5^	3.69 × 10^1^
E80 + 64 mg/L NaCl	−4.38 × 10^2^	7.61 × 10^8^	3.39 × 10^1^

**Table 6:** Qualifying criteria for fracture toughness (*J_IC_*) for MAS in E80 fuel ethanol environment. B: specimen thickness; $b_{o}:$ un-cracked ligament; ${\frac{dJ}{da}|}_{\Delta a_{Q}}$: tearing slope at critical crack extension.

Environmental	Temperature	σ_o_	*J*_0.2_	B	$b_{o}$	$10\;J_{0.2}/\sigma_{o}$	${\frac{dJ}{da}|}_{\Delta a_{Q}}$
Condition	(°C)	(MPa)	(kJ/m^2^)	(mm)	(mm)		
E80 + 0 mg/L NaCl	27	379	435	7.94	9.14	11.48	0.85
E80 + 32 mg/L NaCl	27	379	306	8.00	9.06	8.07	0.97
E80 + 64 mg/L NaCl	27	379	250	7.94	9.15	6.60	0.74

## Abbreviations

The abbreviations used in this manuscript include:

API: American Petroleum Institute
ASTM: American Standard for Testing Materials
$A_{pl\left(i\right)}$: Instantaneous area under the load-plastic load line displacement curve in fracture toughness test
$a_{i}$, $a_{oq}$$\Delta a_{Q}$: Instantaneous crack length, original crack length, crack extension
*B*, *B_N_*: Specimen thickness, Net specimen thickness
$b_{o},$$b_{\left(i-1\right)}$: Un-cracked ligament, at the start of test and at (i − 1)th step
COD: Crack opening displacement
CTOD: Crack tip opening displacement
${\frac{dJ}{da}|}_{\Delta a_{Q}}$: Tearing slope at critical crack extension
*E*: Elastic modulus
EDM: electro-discharge machining
EPFM: Elastic-plastic fracture mechanics
*e_u_*, *e_T_*: Uniform elongation, Total elongation
*H_v_*: Vickers hardness
*J_0.2_*, *J_IC_*, *J_pl_*, *J_str_*: An energy based fracture parameter determined at 0.2 mm crack extension, qualified as plane strain fracture toughness, plastic part of fracture toughness, fracture toughness measured from stretch zone
*K_i_*: Instantaneous stress intensity factor
*K_ISCC_*: Threshold stress intensity factor for SCC
LLD: Load-line displacement
MAS: Micro-alloyed steel
*n*: Strain hardening exponent
N-SSR: Notched- slow strain rate
OCP: Open circuit potential
*Pi*: Instantaneous load
RFA: Renewable fuels association
*S*: Specimen span
SCC: Stress corrosion cracking
SCE: Saturated calomel electrode
SEM: Scanning electron microscope
SFGE: Simulated fuel grade ethanol
SSRT: Slow strain rate testing
SZW: Stretch zone width
TPB: Three-point bend
W: Specimen width
*∆K*: Stress intensity factor range
$\sigma_{o}$, σ_YS_, σ_UTS_: Flow stress, Yield stress, Ultimate tensile stress
ν: Poisson’s ratio
